# The efficacy and safety of crizotinib in the treatment of anaplastic lymphoma kinase-positive non-small cell lung cancer: a meta-analysis of clinical trials

**DOI:** 10.1186/1471-2407-14-683

**Published:** 2014-09-19

**Authors:** Haili Qian, Feng Gao, Haijuan Wang, Fei Ma

**Affiliations:** State Key Laboratory of Molecular Oncology, Cancer Institute/Hospital, Peking Union Medical College & Chinese Academy of Medical Sciences, Beijing, China; Department of Medical Oncology, Cancer Institute/Hospital, Peking Union Medical College & Chinese Academy of Medical Sciences, Beijing, China; Health Division of Guard Bureau, General Staff Department of Chinese PLA, Beijing, China

**Keywords:** Meta-analysis, Crizotinib, Anaplastic lymphoma kinase (ALK), Non-small cell lung cancer (NSCLC)

## Abstract

**Background:**

Crizotinib was granted accelerated approval by the Food and Drug Administration in 2011 for the treatment of anaplastic lymphoma kinase (ALK)-positive non-small cell lung cancer (NSCLC). To evaluate the efficacy and safety of crizotinib, we performed a meta-analysis of published clinical trials using the random effect model.

**Methods:**

The efficacy and safety of crizotinib was evaluated based on 1-year overall survival (OS), progression-free survival (PFS), overall response rate (ORR), partial response, complete response, stable disease, and dose reduction or cessation because of crizotinib toxicity.

**Results:**

Six clinical trials were included in the meta-analysis. Crizotinib treatment demonstrated a 1-year OS of 66.8% (95% CI, 52.2–78.8%) and a PFS of 8.6 months (95% CI, 7.3–9.9 months). The aggregate ORR, partial response and complete response rates were 61.2%, 59.8% and 1.5%, respectively. The proportion of patients achieving stable disease was 42.6% (95% CI, 17.3–72.5%). The most frequently reported adverse effects of crizotinib were mild visual disturbances, nausea, vomiting, diarrhea, constipation, edema, reduction in glomerular filtration rate, and generally reversible but sometimes severe elevations in aspartate aminotransferase and alanine aminotransferase. The proportion of patients who required dose reduction or cessation because of crizotinib toxicity was 6.5% (95% CI, 4.1–10.1%).

**Conclusions:**

This meta-analysis revealed extended survival and improved response rates in patients treated with crizotinib. As a novel, targeted anticancer agent, crizotinib appears to be a favorable treatment option for patients with locally advanced or metastatic ALK-positive NSCLC.

## Background

Lung cancer, of which approximately 85–90% of cases are non-small cell lung cancer (NSCLC), is the most common fatal malignancy among all cancers worldwide, and its incidence has gradually increased over recent decades, [1, 2]. Patients usually present with unresectable locally advanced (stage IIIB) or distant metastases (stage IV) NSCLC. For patients newly diagnosed with advanced NSCLC, the median overall survival (OS) with platinum-based chemotherapy is 7.4–9.9 months, and the median OS with combined chemotherapy and bevacizumab is 12.5 months. Median progression-free survival (PFS) with second-line chemotherapy, such as pemetrexed and docetaxel, is approximately 2.2–2.9 months. Although associated with a higher response rate, OS with gefitinib is similar to that of standard carboplatin plus paclitaxel chemotherapy [3]. The 5-year survival rate of NSCLC is lower than 20% [4, 5]. However, despite the relatively poor prognosis for NSCLC patients, the development of treatments for NSCLC has not yet kept pace [6].

In recent years, the identification of genetic abnormalities that may underlie oncogenic development and progression have revolutionized oncology research [7]. A translocation in the gene encoding the receptor tyrosine kinase anaplastic lymphoma kinase (ALK), leading to the expression of ALK fusion proteins, was first reported in NSCLC patients in 2007 [8, 9]. The activated ALK fusion proteins result in aberrant ALK signaling and oncogenic transformation through several molecular signaling pathways, including PI3K/AKT/mTOR, JAK/STAT, and RAS/MEK/ERK [10]. Constitutive ALK signaling mediates enhanced cell proliferation, cell survival, and metabolism. ALK gene rearrangements are found in approximately 2–7% of unselected patients with NSCLC [11]. Because of the role of ALK in oncogenesis, tyrosine kinase inhibition has been investigated as a therapeutic approach [11]. Crizotinib is a small-molecule selective inhibitor of ALK and mesenchymal epithelial growth factor (c-Met)/hepatocyte growth factor receptor (HGFR) kinases [12]. It was approved via accelerated drug approval by the US Food and Drug Administration in 2011, based on the findings of two early phase clinical trials demonstrating prolonged progression-free survival (PFS; 6–10 months) and high response rates (50–57%) in patients with ALK-positive NSCLC [13, 14]. A further phase 3 clinical trial in patients with ALK-positive NSCLC confirmed the advantage of crizotinib in survival, response rates, and duration of response in ALK-positive patients with NSCLC, although mild to severe adverse effects, commonly reported as gastrointestinal disturbances (nausea, diarrhea, vomiting, and constipation), visual disturbances and fatigue, were noted [15, 16].

This study systematically combines data from published clinical trials to evaluate the efficacy and safety of crizotinib in the treatment of ALK-positive NSCLC using a random effect model following the Preferred Reporting Items for Systematic Reviews and Meta-Analyses (PRISMA) guidelines [17].

## Methods

### Search strategy for the identification of studies

To evaluate the efficacy and safety of crizotinib in the treatment of ALK-positive NSCLC, PubMed, Embase, and the Cochrane Library (all from 1980 to Nov 2013) were searched to identify clinical trials in English-language journals. The search terms used were “crizotinib”, “non-small cell lung cancer”, “carcinoma”, “anaplastic lymphoma kinase”, and “clinical trial” in various combinations. We also manually searched the related references from the bibliography of the selected articles. Only eligible original studies with full text available were selected, and meeting abstracts were excluded. The corresponding authors of some studies were contacted for further information if necessary.

### Article selection criteria

All clinical trials exploring the efficacy and safety of crizotinib in the treatment of ALK-positive NSCLC were considered eligible for the analysis. The inclusion criteria were as follows: (i) articles were clinical trials investigating the efficacy and/or safety of crizotinib in the treatment of ALK-positive NSCLC; (ii) a standardized effect size could be calculated on the evaluations of 1-year overall survival (OS), progression-free survival (PFS), overall response rate (ORR), partial response, complete response, stable disease, and/or dose reduction or cessation because of adverse effects of crizotinib were reported; (iii) articles were in English; and (iv) full text was available. Two investigators (MF and WH) independently assessed the articles for relevancy.

### Data extraction

All articles were first de-identified (article title, author names, journal name and year of publication) before selection. The abstracts of the articles were independently reviewed by two authors (MF and WH). Outcomes were pooled for 1-year OS (defined as the percentage of patients who remained alive 1 year after crizotinib treatment), ORR (defined as the observed alive rate of patients since the date of crizotinib treatment), partial response (defined as the percentage of patients who had a decrease in the size of tumor, or in the extent of cancer in the body, in response to crizotinib treatment), complete response (defined as the percentage of patients who had the disappearance of all signs of cancer in response to crizotinib treatment), dose reduction or cessation because of adverse effects of crizotinib, and mean duration of PFS and stable disease. Data were filtered and transferred into a standard electronic form. Any discrepancies were resolved by discussion until a consensus was reached. If not, the principal investigator (QH) made the final decision on the eligibility of the study and data extraction.

### Statistical analysis

Data management and analysis were performed using Comprehensive Meta-analysis Version 2 (Biostat, Englewood, NJ, USA). Data were pooled statistically using the event rates calculated for primary (the 1-year OS, ORR, partial response, complete response) and secondary endpoints (dose reduction or cessation because of crizotinib adverse effects, and mean duration of PFS and stable disease).

A random effect meta-analysis was conducted to investigate the efficacy and safety of crizotinib in the treatment of ALK-positive NSCLC in this study. Because fixed effects models in meta-analysis assume that one true effect exists, which all studies are estimating, they increase the risk of a Type I error and create overly narrow confidence intervals. In contrast, random effects models assume that true effects are different across studies owing to heterogeneity of patients, treatments or other factors. Random effects models create wider confidence intervals and minimize the risk of Type I error. Therefore, random effects models are recommended to be routinely employed in meta-analysis, particularly when study heterogeneity is expected or found [18].

The measure of heterogeneity was evaluated using Cochran’s Q-test. Additionally, heterogeneity was assessed using the I^2^ statistic. A high value for I^2^ indicates heterogeneity. Publication bias was evaluated using Egger’s test.

### Ethical approval

The study was approved by the Ethics Committee of the Cancer Hospital of Chinese Academy of Medical Sciences.

## Results

**Figure 1:**
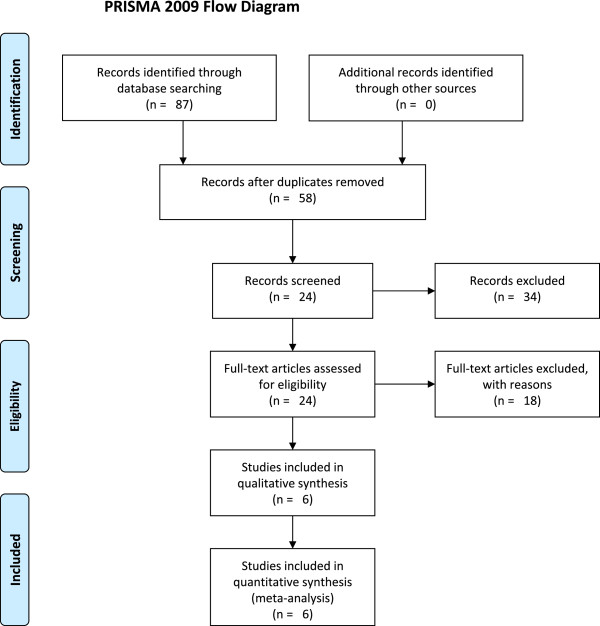
**Flow chart describing the article selection process.**

A total of 87 abstracts were initially selected through database searching, and 63 articles were excluded because they failed to meet the criteria. For the remaining 24 articles, five had the same data presented in other studies, six did not provide sufficient information to calculate an effect size, and seven studies were case reports. The article selection process is shown in Figure 1, and details of the six articles [14, 19–23] selected for our analysis are shown in Table 1.

**Figure 2:**
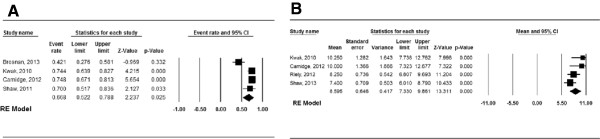
**Forest plot for the aggregate survival data of crizotinib in the treatment of patients with locally advanced or metastatic anaplastic lymphoma kinase (ALK)-positive non-small cell lung cancer (NSCLC). (A)** 1-year overall survival (OS); **(B)** progression-free survival (PFS).

**Figure 3:**
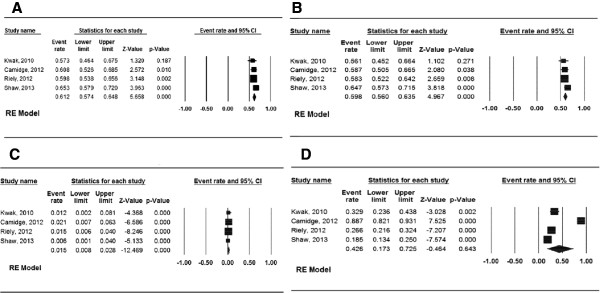
**Forest plot for the aggregate response data of crizotinib in the treatment of patients with locally advanced or metastatic anaplastic lymphoma kinase (ALK)-positive non-small cell lung cancer (NSCLC). (A)** overall response rate (ORR); **(B)** partial response; **(C)** complete response; **(D)** stable disease.

**Figure 4:**
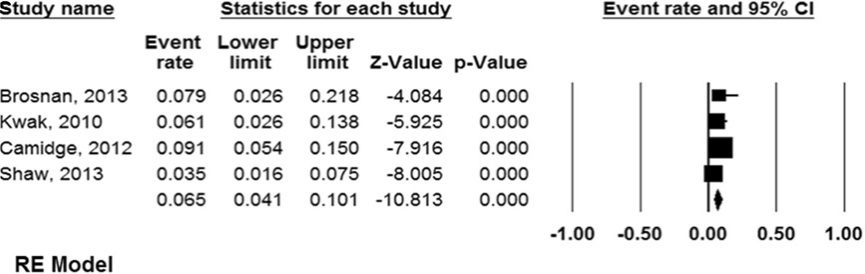
**Forest plot for the aggregate proportion of patients who required dose reduction or cessation because of crizotinib toxicity in the treatment of locally advanced or metastatic anaplastic lymphoma kinase (ALK)-positive non-small cell lung cancer (NSCLC).**

In these 6 studies, the efficacy and toxicity of crizotinib was investigated in the treatment of ALK-positive NSCLC. Analysis of pooled data revealed a 1-year OS of 66.8% (95% CI, 52.2–78.8%; Figure 2A) and a PFS of 8.6 months (95% CI, 7.3–9.9 months; Figure 2B). In terms of response rates, the aggregate ORR (Figure 3A), partial response (Figure 3B) and complete response (Figure 3C) were 61.2% (95% CI, 57.4–64.8%), 59.8% (95% CI, 56.0–63.5%) and 1.5% (95% CI, 0.8–2.8%), respectively. The proportion of patients achieving stable disease (Figure 3D) was 42.6% (95% CI, 17.3–72.5%). The proportion of patients who required dose reduction or cessation because of crizotinib toxicity was 6.5% (95% CI, 4.1–10.1%, Figure 4).

**Table 1:** **Main characteristics of the selected studies**

Citation	Mean age (years)	Number of patients	Duration of follow up (month)	Dose and frequency of crizotinib administration	Trial phase	Tumor histologic type (Adenocarcinoma, %)	Extent of disease (Metastatic, %)
Shaw et al. [21]	50.0	173	12.2	250 mg twice daily	Phase 3	95	95
Brosnan et al. [22]	54.7	38	16.3	250 mg twice daily	NA	NA	NA
Riely [23]	53.0	261	12.0	250 mg twice daily	Phase 2	92	NA
Camidge et al. [19]	52.0	149	16.3	250 mg twice daily	Phase 1	97	NA
Shaw et al. [20]	51.0	56	18.0	250 mg twice daily	Phase 1	96	89
Kwak et al. [14]	51.0	82	6.4	250 mg twice daily	Phase 1	96	NA

NA = not applicable.

There were statistically significant differences for 1-year OS (Q = 14.903, *p* = 0.002, I^2^ = 79.870) and stable disease (Q = 122.520, *p* <0.001, I^2^ = 97.551). No significant difference was observed for other outcomes (*p* >0.05). Funnel plots and Egger’s regression test revealed no significant publication bias (*p* >0.05).

## Discussion

The goal of this meta-analysis was to evaluate the efficacy and safety of crizotinib in the treatment of ALK-positive NSCLC. The aggregated effect size revealed that crizotinib treatment shows generally extended survival (1-year OS: 66.8%; PFS: 8.6 months) and improved response rates (ORR: 61.2%; partial response: 59.8%; complete response: 1.5%; stable disease: 42.6%). These findings strongly indicate the effectiveness of crizotinib treatment in patients with ALK-positive NSCLC. In a retrospective analysis of a phase 1 trial [20], ALK-positive patients who received crizotinib (n = 82) showed improved survival compared to ALK-positive control patients (n = 36) who did not receive crizotinib. In the second- and third-line settings, 1-year OS was 70% versus 44%, respectively; and 2-year OS was 55% versus 12%, respectively. The effect of crizotinib treatment (n = 173) was also compared with standard-of-care NSCLC treatments (docetaxel or pemetrexed as a single agent, n = 174) in ALK-positive patients with advanced NSCLC previously treated with one platinum-containing regimen in a Phase 3 trial [21]. Results showed that PFS was prolonged in the crizotinib-treated group (7.7 months vs. 3.3 months, *p* <0.0001). Patients in the crizotinib arm completed more treatment cycles than those in the standard chemotherapy arm. Response was significantly improved (65% vs. 20%, *p* <0.0001). Thus, crizotinib treatment demonstrated an improvement in survival and response rates, which were superior to standard-of-care chemotherapy. As a novel targeted anticancer agent, crizotinib appears to be a favorable treatment option for patients with locally advanced or metastatic ALK-positive NSCLC.

Notably, 6.5% of patients have to reduce the dose or discontinue crizotinib treatment because of toxicity. In the studies included in this meta-analysis, the most frequently reported adverse effects were mild visual disturbances, nausea, vomiting, diarrhea, constipation, edema, reduction in glomerular filtration rate, and generally reversible but sometimes severe elevations in aspartate aminotransferase and alanine aminotransferase. Shaw et al. [21] found that adverse events reported with crizotinib treatment of ALK-positive NSCLC patients were comparable to docetaxel or pemetrexed, with similar severe (grade 3 or 4) reactions across the treatment groups. Discontinuation rates were slightly higher in the chemotherapy arm (10%) compared with the crizotinib arm (6%). Although generally tolerated, the toxicity of crizotinib should be monitored in order to maximize its safety [24, 25], and the dose of crizotinib needs to be adjusted when necessary [26].

As a newly approved medication, evidence of the efficacy and safety of crizotinib in the treatment of ALK-positive NSCLC is relatively incomplete. The findings from this meta-analysis are therefore valuable for physicians and public health policy makers in formulating strategies to maximize the efficacy and minimize crizotinib toxicity. However, given the relatively small numbers of cases included into the trials, generalization of the conclusions from this study to all patients with ALK-positive NSCLC should be cautious.

## Conclusions

In conclusion, the meta-analysis investigated the efficacy and safety of crizotinib in the treatment of patients with locally advanced or metastatic ALK-positive NSCLC. Clinical trials with crizotinib treatment show extended survival and improved response rates, along with tolerable toxicity. As a novel targeted anticancer agent, crizotinib appears to be a favorable treatment option for patients with locally advanced or metastatic ALK-positive NSCLC. Further blinded, placebo-controlled studies with larger sample sizes are needed to compare the efficacy and safety of crizotinib with other NSCLC treatments.
